# Comparative effect of driving side on low back pain due to Repetitive Ipsilateral Rotation

**DOI:** 10.12669/pjms.35.4.488

**Published:** 2019

**Authors:** Syed Asadullah Arslan, Mohammad Reza Hadian, Gholamreza Olyaei, Saeed Talebian, Mir Saeed Yekaninejad, Mir Arif Hussain

**Affiliations:** 1 Syed Asadullah Arslan, DPT, PhD, Department of Physiotherapy, School of Rehabilitation, Tehran University of Medical Sciences, Tehran, Iran; 2 Mohammad Reza Hadian, PhD, Department of Physiotherapy, School of Rehabilitation, Tehran University of Medical Sciences, Tehran, Iran; 3 Gholamreza Olyaei, PhD, Department of Physiotherapy, School of Rehabilitation, Tehran University of Medical Sciences, Tehran, Iran; 4 Saeed Talebian, PhD, Department of Physiotherapy, School of Rehabilitation, Tehran University of Medical Sciences, Tehran, Iran; 5 Mir Saeed Yekaninejad, PhD, Department of Physiotherapy, School of Rehabilitation, Tehran University of Medical Sciences, Tehran, Iran; 6 Mir Arif Hussain, M.Sc.PT, Department of Physiotherapy, School of Rehabilitation, Tehran University of Medical Sciences, Tehran, Iran

**Keywords:** Lower back pain, Occupational health, Prevalence, Risk factors, Sacroiliac joint

## Abstract

**Objective:**

To determine the effects of repetitive ipsilateral rotation on low back pain among the taxi drivers of right and left hand drive.

**Methods:**

A total of 1200 (600 Iran+600 Pakistan) male taxi drivers, aged between 20-60 years with work experience of more than one year were randomly selected and interviewed in Tehran (Iran) & Lahore (Pakistan) to fill self-administered questionnaires in Persian and Urdu languages which contained socio-demographic, work related and LBP characteristics. Chi-square test and multiple logistic regression models were employed for statistical analyses.

**Results:**

Point, one week, one year and lifetime prevalence of LBP among right hand drive taxi drivers was 26.7%, 35.5%, 49.8% and 77.7% respectively. Point, one week, one year and lifetime prevalence of LBP among left hand drive taxi drivers was 37%, 42.7%, 53.5% and 72.3% respectively. Mean Numeric Pain rating scale (NPRS) score was 4.15 (SD=1.42) in Pakistan, while in Iran it was 4(SD=1.57). There was no significant difference regarding pain intensity (p=0.123) between drivers from both countries. Mean Roland-Morris Questionnaire (RMQ) score among drivers in Pakistan with LBP was 7.76(SD= 2.50), while in Iranian drivers who had LBP, mean RMQ score was 7.71(SD=2.99).

**Conclusion:**

Static or less dynamic muscles are more prone to LBP due to lower endurance. Lack of exercising habit, work as a driver for more number of years, driving within city, more driving hours in a day, forward bending, lifting, no seat comfort, lack of awareness regarding ergonomics and lower satisfaction level of job were the main reasons of LBP.

## INTRODUCTION

Globally, LBP is the leading cause of disability which is increasing in low and middle-income countries during past few decades.^1^ It is the leading cause of activity limitation and work absence all over the world with the point prevalence ranging from 19% to 39%.^2^ Work-related lower back pain (WLBP) due to lifting, twisting, prolonged sitting or standing, reaching at or above shoulder level, and working in an awkward position is very common.^3^ Seated workers such as vehicle drivers frequently experience LBP caused by long hours of driving in a restricted posture, car vibration or shocks from roads.^4^ Taxi drivers always remain under stress due to prolonged and consecutive work, working during day & night shifts, irregularity in work time and poor physical condition. Environmental factor (heat & cold, poor lighting), sitting place, uniform, repetitive trauma, job insecurity (insurance, social support and family deprivation) and other factors like poor road conditions are also important factors which affect the overall health condition of drivers.^5^ Previous studies have reported LBP disorder associated with driving in the developed^6^,^7^ and developing countries, such as India.^8^

This study was conducted in Tehran, the capital city of Islamic Republic of Iran which has left hand drive and in Lahore-Pakistan where the driving side is on right. Taxi drivers in Lahore and Tehran are facing LBP problem due to different factors under different cultural influence. No study has reported LBP among taxi drivers in Iran and Pakistan, therefore this was the first study which has discussed LBP and its associated factors due to driving in these two left & right hand drive countries. Second aim of this study was to check any difference in the effects of associated factors on LBP among drivers belonging to two different races and cultures.

## METHODS

This cross-sectional study was approved by the ethical committee of Tehran University of Medical Sciences (TUMS). A total of 1200 (600 Iran+600 Pakistan) male taxi drivers, aged between 20-60 years with work experience of more than one year were randomly selected. After having their consent, the researcher interviewed the drivers in Tehran (Iran) & Lahore (Pakistan) to fill self-administered questionnaires in Persian and Urdu languages which contained socio-demographic, work related and LBP characteristics. Previous studies^9^ have reported the self-reporting, a relatively reliable and valid method to assess the time spent on motor vehicle driving. Drivers with lower limb radiculopathy, postural dysfunction or limb length discrepancy, history of surgery, trauma or involved in any pathological disease were excluded. Numeric Pain rating scale (NPRS) was used to rate the pain intensity from 0 (“no pain”) to 10 (“worst possible pain”).^10^

For analysis of the data, means and standard deviations for numerical variables, frequency and percentage for grouping variables were used. Chi-squared test was used to find relation between LBP and work-related risk factors. Association of LBP prevalence with possible risk factors was predicted with the help of multiple logistic regression analysis and results were represented by odds ratios (OR) along with the 95% confidence interval (CI). A P-value <0.05 was considered as statistically significant. All statistical tests were performed two sided.

## RESULTS

The mean age of the participants from Pakistan was 42.82 (SD= 9.29) years, while in Iran it was 47.37 (SD=8.90). The independent-samples t-test showed strong significant difference between the age groups in both countries (p<0.001). Mean weight of the subjects was slightly higher in Iran (81.94 kg, SD=10.73) compared to Pakistan (80.43, SD=10.77) and statistically it had significant difference (*P*=0.016). Mean height was 172.15 cm, SD=6.06 in Iran, while in Pakistan it was 171.54 cm, SD=6.38, but statistically there was no association between two height groups (p =0.091). Mean body mass index of Pakistani drivers was 27.34 (SD=3.42) and it was 27.65 (SD=3.42) in Iranian drivers. Association of demographics, personal and work related characteristics with LBP has been shown in Table-I.

**Table I:** Demographic, personal and work ergonomic characteristics of drivers.

Variables	LBP in Pakistan			LBP in Iran		

	Yes N (%)	No N (%)	P-value	Yes N (%)	No N (%)	P-value
*Marital status*						
Single	7(16.3)	36(83.7)	0.073	9(13.4)	58(86.6)	<0.001
Married	153(27.5)	404(72.5)		213(40)	320(60)	
*Education Level*						
Primary	79(35.6)	143(64.4)	0.001	49(47.6)	54(52.4)	<0.001
High School	60(23.2)	199(76.8)		99(44.2)	125(55.8)	
Intermediate	16(20.3)	63(79.7)		68(28.3)	172(71.7)	
Graduation	5(12.5)	35(87.5)		6(18.2)	27(81.8)	
*Exercise*						
No	135(30.9)	302(69.1)	<0.001	184(50.8)	178(49.2)	<0.001
Yes	25(15.3)	138(84.7)		38(16)	200(84)	
*Stretching Exercise*						
No	134(28.5)	336(71.5)	0.057	161(46.3)	187(53.7)	<0.001
Yes	26(20)	104(80)		61(24.2)	191(75.8)	
*Sleep Disturbance*						
No disturbance	27(8.6)	286(91.4)	<0.001	120(26.4)	334(73.6)	<0.001
1-2x/week	36(31)	80(69)		28(60.9)	18(39.1)	
3x/week	49(47.1)	55(52.9)		22(68.8)	10(31.2)	
No Sleep	48(71.6)	19(28.4)		52(76.5)	16(23.5)	
*Smoking Habit*						
No	59(20.3)	231(79.7)	<0.001	108(34.1)	209(65.9)	0.068
Yes	101(32.6)	209(67.4)		114(40.3)	169(59.7)	
*Job Type*						
Self- Employee	98(31.9)	209(68.1)	0.003	201(41.2)	287(58.8)	<0.001
Work for company	62(21.2)	231(78.8)		21(18.8)	91(81.2)	
*Driving Area*						
Within City	113(29.2)	274(70.8)	0.059	179(41.1)	257(58.9)	<0.001
Out of city	47(22.1)	166(77.9)		43(26.2)	121(73.8)	
*Working hours/ Day*						
≤ 8hrs	36(19.6)	148(80.4)	0.009	17(15.7)	91(84.3)	0.005
>8hrs	124(29.8)	292(70.2)		205(41.7)	287(58.3)	
*Work Experience*						
≤5 Years	18(17)	88(83)	0.012	8(16)	42(84)	<0.001
6-10 years	12(20)	48(80)		29(23.6)	94(76.4)	
≥10 years	130(30)	304(70)		185(43.3)	242(56.7)	
*Second Job*						
No	145(28)	372(72)	0.056	208(38.4)	334(61.6)	0.033
Yes	15(18.1)	68(81.9)		14(24.1)	44(75.9)	
*Previous Job*						
No	126(35)	234(65)	<0.001	142(51.1)	136(48.9)	<0.001
Yes	34(14.2)	206(85.8)		80(24.8)	242(75.2)	
*Number of Kilometers /Day*						
≤100 km	60(21.3)	222(78.7)	0.005	33(20.6)	127(79.4)	<0.001
≥100 km	100(31.4)	218(68.6)		189(43)	251(57)	
*Forward bending*						
No	90(21.2)	335(78.8)	<0.001	96(28.7)	238(71.3)	<0.001
Yes	70(40)	105(60)		126(47.4)	140(52.6)	
*Weight lifting*						
No	30(19.9)	121(80.1)	0.029	23(18.5)	101(81.5)	<0.001
Yes	130(29)	319(71)		199(41.8)	277(58.2)	
*Weight lifting frequency*						
Seldom	50(19.9)	201(80.1)	<0.001	59(36.2)	173(55.4)	<0.001
Often	80(40.4)	118(59.6)		139(44.6)	101(80.8)	
*Seat Adjustment*						
No	111(27.5)	293(72.5)	0.294	172(38.8)	271(61.2)	0.071
Yes	49(25)	147(75)		50(31.8)	107(68.2)	
*Ergonomics Awareness*						
No	88(20.8)	336(79.2)	<0.001	114(41.2)	163(58.8)	0.052
Yes	72(40.9)	104(59.1)		108(33.4)	215(66.6)	
*Seat Comfortable*						
No	44(37.6)	73(62.4)	0.003	90(54.9)	74(45.1)	<0.001
Yes	116(24)	367(76)		132(30.3)	304(69.7)	
*Back Support at Seat*						
No	125(25)	376(75)	0.024	168(38.6)	267(61.4)	0.107
Yes	35(35.4)	64(64.6)		54(32.7)	111(67.3)	

Point, one week, one year and lifetime prevalence of LBP in Pakistan was 26.7%, 35.5%, 49.8% and 77.7% respectively. Point, one week, one year and lifetime prevalence of LBP in Iran was 37%, 42.7%, 53.5% and 72.3% respectively. Mean pain intensity based on NPRS was 4.15 (SD=1.42) in Pakistan, while in Iran it was 4(SD=1.57). There was no significant difference regarding pain intensity (p=0.123) between drivers from both countries. LBP related disability among the drivers was assessed by Roland Morris Disability Questionnaire (RMQ), which has 24-items ranging from 0 to 24. Items are scored 0 if left blank or 1 if endorsed; higher scores represent higher levels of pain-related disability.^11^ Mean RMQ score among drivers in Pakistan with LBP was 7.76(SD= 2.50), while in Iranian drivers who had LBP, mean RMQ score was 7.71(SD=2.99). Statistically, there was no significant difference in the RMQ scores of two populations (P=0.774).

Multiple logistic regressions were used to predict higher probability of LBP occurrence among drivers and results of important occupational risk factors are shown in tables (Table II & III).

**Table II:** Logistic regression model predicting the association of occupational risk factors for LBP in Pakistan.

Factors	OR (adjusted)	95% CI	P-value
*Exercise*			
No (Reference)			
Yes	0.493	0.273-.888	0.019
*Sleep Disturbance*			
No Disturbance (Reference)			
1 to 2 times/Week	4.832	2.63-8.89	<0.001
3 times / Week	10.887	5.88-20.16	
More than 3 times/ Week	27.950	13.46-58.10	
*Smoking*			
Yes	2.45	1.5-4.01	<0.001
No (Reference)			
*Type of job*			
Self-Employee	2.02	1.18-3.47	0.010
Work for company (Reference)			
*Driving Area*			
Within City (Reference)			
Out of City	2.19	1.18-4.10	0.013
*Weight Lifting*			
No (Reference)			
Yes	2.32	1.26-4.27	0.007

**Table III:** Logistic regression model predicting the association of occupational risk factors for LBP in Iran.

Factors	OR (adjusted)	95% CI	P-value
*Exercise*			
No			
Yes (Reference)	12.23	6.58- 22.74	<0.001
*Sleep Disturbance*			
No Disturbance (Reference)			
1 to 2 times/Week	5.56	2.41-12.83	<0.001
3 times / Week	12.74	3.46-46.92	
More than 3 times/ Week	14.35	5.67-36.32	
*Smoking*			
Yes	2.14	1.22-3.78	0.008
No (Reference)			
*Type of job*			
Self-Employee	15.14	6.46-35.46	<0.001
Work for company (Reference)			
*Driving Area*			
Within City	2.98	1.62-5.49	<0.001
Out of City (Reference)			
*Weight Lifting*			
No (Reference)			
Yes	6.63	2.89-15.23	<0.001

## DISCUSSION

Pakistan is the 7th while Iran is the 17th populous country in the world. Iran has made remarkable progress in the health sector during the last 20 years but health and social indicators in Pakistan remained low.^12^,^13^ Despite better health system, present study showed higher prevalence of LBP among Iranian taxi drivers which might be due to genetic, cultural and specific ergonomic environmental differences between two nations. According to the National Health Survey in Iran, LBP among the Iranian society was 29.3%^14^ which is less than the LBP among taxi drivers in Iran as reported in present study. Previous studies have also reported that LBP frequency among drivers was 1.6-2.0 times the reference prevalence in that society.^15^ Similar to present study, (26.7% Pakistan, 37% Iran), high prevalence of LBP was also reported by other researchers.^2^,^6^,^15^ Other researchers reported average Visual Analogue Scale (VAS) score of 4.3 among LBP drivers which is similar to the present study.^4^

Different predisposing demographic factors for LBP like marital status, low education level, lack of exercise, sleep disturbance, smoking and more work experience remained common for working population.^16^ Depression and health habits may have indirect influence on marital life but direct influences of cardiovascular, endocrine, immune, neurosensory, and other physiological mechanisms have been reported by previous studies^17^ and in present study, higher LBP prevalence among married drivers is also consistent with the “National Health Survey” in Iran.^14^ Education can have substantial effects on lifestyles, such as smoking, exercise and diet. Similar to present study, numerous studies have documented that higher education is positively associated with better health throughout the lifespan.^18^ Smoking increases the risk of LBP; biologic studies have indicated that smoking causes intervertebral disc degeneration and decreases bone mineral density in the lumbar spine which could be the reason of higher prevalence of LBP among smoker drivers.^19^ In present study, lower prevalence of LBP among drivers showed statistically significant association with exercising habit. Henchoz and Kai-Lik has reported that targeted exercises can improve spinal muscles coordination and functional disability could be avoided by stabilizing lumbar.^20^ Another promising finding was significant association (p<0.001) between LBP and sleep disturbance among drivers from both countries which ties well with previous studies wherein, the relationship between sleep disturbance and pain was reported as reciprocal, such that pain disturbs sleep continuity/quality and poor sleep further exacerbates pain.^21^

Factors such as driving area, working hours & number of kilometers in a day, job type, weight lifting and driving seat comfortability were the specific risk factors causing LBP among taxi drivers in Pakistan & Iran and results of present study are similar to the previous such studies.^6^,^7^,^10^ More working hours (hrs) & number of kilometers in a day, definitely cause postural strains on back muscles and exposure to whole-body vibration.^15^ Researchers like Porter and Gyi similar to present study also found that driving more than 20 hours a week was associated with high frequency of LBP. Their results were also in good agreement with present study regarding job type, seat adjustment and seat type; those individual who drove cars as part of their job face more LBP and drivers who had adjustable lumbar support experience less episodes of LBP.^22^ Exposure to biomechanical strains such as bending, twisting and weight lifting also put the driver at higher risk of LBP. Results obtained in present study are broadly consistent with the previous studies while showing significant association (p<0.05) between lifting and bending with LBP.^15^

Few factors showed different effects on the drivers of these two countries. Contrary to results from Iran, marital status did not show significant association with LBP in Pakistan. Post marriage body physiology might be one reason and results from the “National Health survey in Iran” also showed significant association (p<0.001) between LBP and marital status.^14^ Stretching exercise had shown weak significant association with LBP in Pakistan and only 130(21.67%) drivers had the habit of doing stretching exercises while in Iran, 252(42%) drivers used to perform stretching exercises and had significant association with LBP. Data from three national surveys among Iranian adults have shown that more than 80% of the Iranian population is physically inactive.^23^ In Tehran, 16 million vehicular trips take place every day with the average speed of 15 km/h during peak hours in the Central Business District.^24^ Drivers use clutch and brakes repetitively due to heavy traffic which might put extra pressure on SIJ in sitting position and cause LBP. In Iran most of the drivers drove their taxis within city and are 2.98 times at greater risk of LBP than out of city drivers while in Pakistan, those who drove out of city are at greater risk (2.19 times) (Tables II & III). Lahore has more than three million vehicles on its roads; but the number of taxi cars is less.^25^ Therefore, LBP was high among the drivers from Tehran and the results were significant too. Regression model in present study has also predicted higher risk of LBP for Iranian drivers (15.14 times) compared to Pakistani drivers (2.02) because more drivers in Tehran were self-employed (Tables II & III). In Tehran, more number of drivers 440(73.34%) were traveling distance of ≥100 Km per day and LBP prevalence was higher (43%) than Pakistani drivers (31.4%) who were covering less distance in a day. Similar to present study, previous researchers had also declared the long hours of driving in a restricted posture, a good reason of LBP.^4^

### Limitations of the study

Most of the information was collected while interviewing the drivers which cannot be ascertained. Weak memories of drivers made the few questions doubtful as they were not able to explain exact conditions. We could not specify the duration of each drive and number of rest sessions between different drives in a day. Medical history of drivers before becoming driver was not available, so we could not judge, how much that particular individual was prone to LBP. Available drivers at the taxi stands were interviewed and more number of absent drivers might be suffering from LBP at that particular day.

## CONCLUSIONS

Different demographic and ergonomic factors were causing LBP among taxi drivers of both Pakistan and Iran. Predisposing ergonomic factors had more devastating effects on Iranian drivers due to higher body mass index, traffic congestion in Tehran city, remaining on driving seat for extra duration, covering longer distance per day and repetitive application of clutch and brake in traffic jam in the city.

### Author's Contribution

**SAA** is the Ph.D. student, who carried out the research.

**MRH & GRO** were the supervisors and co-supervisors who helped in write up.

**ST** conceived of the presented idea and helped in methodology.

**MSY** did computational framework and analyzed the data.

**MAH** helped in data collection.

## References

[1] Hartvigsen J, Hancock MJ, Kongsted A, Louw Q, Ferreira ML, Genevay S (2018). What low back pain is and why we need to pay attention. Lancet.

[2] Manchikanti L, Singh V, Falco FJ, Benyamin RM, Hirsch JA (2014). Epidemiology of low back pain in adults. Neuromodulation.

[3] Pransky G, Benjamin K, Hill-Fotouhi C, Fletcher KE, Himmelstein J, Katz JN (2002). Work-related outcomes in occupational low back pain:a multidimensional analysis. Spine.

[4] Miyamoto M, Konno S, Gembun Y, Liu X, Minami K, Ito H (2008). Epidemiological study of low back pain and occupational risk factors among taxi drivers. Ind Health.

[5] Hajiamini Z, Cheraghalipour Z, Marzabadi A, Ebadi A, Norouzi Koushali A (2011). Comparison of job stress in military and non-military drivers in Tehran city. J Mil Med.

[6] Hedberg GE (1988). The period prevalence of musculoskeletal complaints among Swedish professional drivers. Scand J Soc Med.

[7] Schwarze S, Notbohm G, Dupuis H, Hartung E (1998). Dose–response relationships between whole-body vibration and lumbar disk disease—a field study on 388 drivers of different vehicles. J Sound Vib.

[8] Kumar A, Varghese M, Mohan D, Mahajan P, Gulati P, Kale S (1999). Effect of whole-body vibration on the low back:a study of tractor-driving farmers in north India. Spine.

[9] Palmer KT, Haward B, Griffin MJ, Bendall H, Coggon D (2000). Validity of self reported occupational exposures to hand transmitted and whole body vibration. Occup Environ Med.

[10] Ferraz MB, Quaresma M, Aquino L, Atra E, Tugwell P, Goldsmith C (1990). Reliability of pain scales in the assessment of literate and illiterate patients with rheumatoid arthritis. J Rheumatol.

[11] Stratford PW, Riddle DL (2016). A Roland Morris Disability Questionnaire target value to distinguish between functional and dysfunctional states in people with low back pain. Physiother Can.

[12] Mehrdad R (2009). Health system in Iran. Japan Med Assoc J.

[13] Ghaffar A, Kazi BM, Salman M (2000). Health care systems in transition III. Pakistan, Part I. An overview of the health care system in Pakistan. J Public Health Med.

[14] Biglarian A, Seifi B, Bakhshi E, Mohammad K, Rahgozar M, Karimlou M (2012). Low back pain prevalence and associated factors in Iranian population:findings from the national health survey. Pain Res Treat.

[15] Chen J-C, Chang W-R, Chang W, Christiani D (2005). Occupational factors associated with low back pain in urban taxi drivers. Occup Med.

[16] Spyropoulos P, Papathanasiou G, Georgoudis G, Chronopoulos E, Koutis H, Koumoutsou F (2007). Prevalence of low back pain in Greek public office workers. Pain Physician.

[17] Kiecolt-Glaser JK, Newton TL (2001). Marriage and health:his and hers. Psychol Bull.

[18] Eide ER, Showalter MH (2011). Estimating the relation between health and education:What do we know and what do we need to know?. Econ Educ Rev.

[19] Shiri R, Karppinen J, Leino-Arjas P, Solovieva S, Viikari-Juntura E (2010). The association between smoking and low back pain:a meta-analysis. Am J Med.

[20] Henchoz Y, So AK-L (2008). Exercise and nonspecific low back pain:a literature review. Joint Bone Spine.

[21] Smith MT, Haythornthwaite JA (2004). How do sleep disturbance and chronic pain inter-relate?Insights from the longitudinal and cognitive-behavioral clinical trials literature. Sleep Med Rev.

[22] Porter JM, Gyi DE (2002). The prevalence of musculoskeletal troubles among car drivers. Occup Med.

[23] Farmanbar R, Niknami S, Heydarnia A, Hajizadeh E, Lubans DR (2009). Predicting exercise behavior among Iranian college students using the transtheoretical model and structural equation modeling. Eur J Sci Res.

[24] Pojani D, Stead D (2017). The urban transport crisis in emerging economies.

[25] Aslam MJ, Aslam MA, Batool A (2008). Effect of noise pollution on hearing of public transport drivers in Lahore city. Pak J Med Sci.

